# CT anatomy and normal radiography of the skull of the Rhesus monkey (*Macaca mulatta*)

**DOI:** 10.1002/vms3.1213

**Published:** 2023-07-27

**Authors:** Ali Reza Vajhi, Sarang Soroori, Reihaneh Soflaei, Omid Zehtabvar, Seyyed Hossein Modarres Tonekabony, Iman Memarian

**Affiliations:** ^1^ Department of Surgery and Radiology, Faculty of Veterinary Medicine University of Tehran Tehran Iran; ^2^ Veterinary Radiology DVSc Student, Faculty of Veterinary Medicine University of Tehran Tehran Iran; ^3^ Anatomy Sector, Department of Basic Sciences Faculty of Veterinary Medicine University of Tehran Tehran Iran; ^4^ Doctor of Veterinary Medicine Faculty of Veterinary Medicine, University of Tehran Tehran Iran; ^5^ FPWC (Foundation for the Preservation of Wildlife Cultural Assets), IAR (International Animal Rescue), Felid Tag, Deer Tag, Persian Leopard and Cat Specialist Group (IUCN) Veterinary Advisor Tehran Iran

**Keywords:** CT scan, morphometry, radiography, Rhesus monkey, skull

## Abstract

**Background:**

Considering the relationship between human morphology and physiology with the Rhesus monkey, this animal is the most prominent species of laboratory primate for human and animal health research. Moreover, sending *Macaca mulatta* monkey into space and simulating a living environment for humans shows the similarity of this animal's physiology with humans.

**Objective:**

So far, no comprehensive study has been done on computed tomography (CT) scan and radiography of skulls in Rhesus monkeys. Therefore, providing accurate documents from the CT anatomy of the skull in these animals can help us to better understand normal conditions and diseases, and we can use a functional atlas of diagnostic imaging from the skull of this animal.

**Methods:**

Ten mature monkeys weighing 6.5 kg were used for this project (five males and five females). A radiographic examination with standard views was performed during general anaesthesia. Then the monkeys were placed in a spherical CT scan during general anaesthesia with standard sternal recumbency.

**Results:**

The frontal bone was seen as two parallel radiopaque lines coming forward and downward. The frontal sinus in the Rhesus monkey was not visible in both lateral and dorsoventral radiographs, which could indicate the degeneration of this sinus in this species. The number of teeth in an adult monkey was 32. Molar teeth had a bilophodont arrangement.

**Conclusions:**

The comparison between the size of the eye ball in human and Rhesus monkey, unlike other measured parameters, did not differ much, and this indicates that the volume ratio of the eye ball to the whole skull in Rhesus monkey is higher than that of humans.

## Graphical abstract

Considering the relationship between human morphology and physiology with the Rhesus monkey, this animal is the most prominent species of laboratory primate for human and animal health research. In this study, an attempt has been made to provide an anatomical atlas of the skull of this species and compare it with the human species.

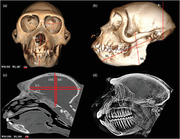

## 1 INTRODUCTION

The Rhesus monkey with the scientific name *Macaca mulatta* is one of the most well‐known species of the monkey family. This species belongs to the subfamily Cercopithecinae, the kingdom Papionini and the genus *Macaque*. There are 22 species in this genus (Ankel‐Simons, 2007). *M. mulatta* has six subspecies, *M. m. brevicauda*, *M. m. lasiota*, *M. m. mulatta*, *M. m. sanctijohannis*, *M. m. vestita* and *M. m. villosa* (Smith & McDonough, 2005).

The Rhesus monkey is brown or grey, and its face is pink and has no hair. Moreover, the tail area of these animals is the same colour. This animal lives an average of 25 years. The size of the tail is between 22.9 and 20.7 cm, and it has an average size among other species (Fooden, 2000). Like other macaque species, males and females in Rhesus are also dimorphic. Normally, the size of the male body is about 53 cm, and the weight is 7.7 kg. The female is smaller, and its body length is about 47 cm, and its weight is 5.34 kg. The age of maturity of this animal is between 3 and 4 years (Kendrick et al., 1962).

Rhesus monkey is diurnal and alternates between water and land. Rhesus lives in a variety of habitats, including wet and dry areas, rainforests and even around human habitations, including temples and roads. Rhesus monkeys are widely acclimated to cohabitation with humans and continue to grow near human habitation, both in rural and urban areas (Choudhury, 2001).

Rhesus is omnivorous, but its main diet consists of fruits. It also feeds on seeds, roots, sprouts and grains. It also feeds on termites, grasshoppers, ants and beetles. When food is limited, it searches for food during the day. The Rhesus monkey has a dedicated sac‐like organ that allows the animal to temporarily store food (Gogoi & Das, 2018).

In 1948, in order to investigate the biological effects of space travel on humans, the United States of America sent the first primate named Albert, who was a Rhesus monkey, into space, 63 km away from the earth. Albert perished during the voyage (Burgess & Dubbs, 2007). Moreover, in 2012, after the first two experiments failed, the Iranian Space Agency launched a Rhesus monkey named Aftab 120 km from the earth by the pioneer probe and successfully returned him to the earth (Gogoi & Das, 2018).

The primate class has unique features in the structure and composition of the bones of the skull, which are more prominent compared to other species. Among these major differences, we can point out the enlargement and increase in the complexity of the brain, which shows itself in the form of an increase in the size of the skull. Another important adaptation of this category is the increase in vision and the ability to see objects in three dimensions. This feature is clear in the position and size of the eye cavity. The relative reduction of the sense of smell is also seen as the apparent reduction of the olfactory region. Some of the functions of the teeth and snout in primates are performed by grasping and pentadactyl hands. The number and size of the teeth and consequently the size of the oral cavity, the angle of the upper jaw, the height of the ascending branch of the upper jaw and its angle with the skull and the strength of this bone due to its thickness are special features of these animals. The inclination of the foramen magnum towards the front indicates a change in the type of walking of this animal, which moves on two legs. This feature is in contrast to the structure of the foramen magnum in the skulls of other mammals, which tend towards the back of the body. Given the similarity of the head between monkeys and humans, we were also encouraged to compare morphometric features in Rhesus monkeys and humans (Ankel‐Simons, 2007).

Skull bones have two types of replacement in terms of evolution; intra‐cartilaginous and intramembranous ossifications. In intramembranous ossification, bone develops on a preformed cartilage matrix, whereas intramembranous ossification is formed at connective tissue junctions without the involvement of a cartilaginous framework (Kealy et al., 2011).

The primate skull has 22 bones (apart from the ear ossicles). Except for the mandible (which makes up the lower jaw), the bones of the skull are connected by sutures, which are fixed fibrous joints, and form the cranium. The cranium itself is divided into three parts:

- An upper dome‐shaped part called the calvaria that covers the cranial cavity containing the brain.
- A base that includes the floor of the cranial cavity.
- A lower anterior part called the facial skeleton or viscerocranium.

The bones forming the calvaria are mainly the unpaired temporal and parietal bones and parts of the unpaired frontal, sphenoid and occipital bones. The bones forming the base of the skull are mainly parts of the sphenoid, temporal and occipital bones. The bones that make up the facial skeleton include the nasal pair, palatine, lacrimal, zygomatic, maxillae and inferior concha of the nose and non‐paired vomer bone. The mandible is neither part of the cranium nor part of the facial skeleton (Kendrick et al., 1962).

The skull bone structures are lighter and at the same time strong due to the cavities and trabecular divisions inside them. These structures are called intracranial sinuses. Primates have sinuses in the frontal region, in the centre of the skull, in the body of the sphenoid bone and in the maxillary region (Rae et al., 2002), in the family Cercopithecinae – except the genus Macaque – they lack this paranasal sinus (Ankel‐Simons, 2007).

Non‐invasive diagnostic imaging methods can provide accurate information about the body, where medical physics, medical engineering and biology are used. To examine bony structures such as the skull, digital radiography can be used. But due to the overlapping of different structures of the skull, it is practically impossible to evaluate the details of bone components in many cases. Moreover, the examination of small fractures caused by pressure or impact may be not seen well in this method due to the two‐dimensionality of the image and overlapping structures, so a computed tomography (CT) scan is the preferred method. However, valuable information is obtained from the radiograph, and certain anatomical areas, such as the temporomandibular joint, teeth and frontal sinuses, can be better evaluated in the radiograph with oblique views (Kealy et al., 2011).

X‐rays were discovered in 1895 by Roentgen, a professor at the University of Wurzburg, Germany, who was investigating and experimenting with cathode rays in a Crooks tube. By substituting the photographic plate instead of the fluorescent glass, Roentgen was able to obtain a figure of the bones of his wife's hand using X‐ray (Thrall, 2018). Several radiographic views can be used to examine the different structures of the skull. Basic views include lateral, dorsoventral, or ventral–dorsal, left or right lateral oblique, anterior–posterior and intraoral views. It is easier to project the dorsoventral view, but in this position, the skull is further away from the film and may be enlarged, although this is not clinically significant (Kealy et al., 2011).

CT scan is a non‐invasive method that uses X‐rays to create a cross‐sectional image of the body without making a surgical incision. In this method, valuable information is obtained from different parts of the body with minimal risk. CT scan provides useful information by providing accurate cross‐sectional images of the nasal cavity, paranasal sinuses and brain cavity. Moreover, a CT scan provides a higher level of soft tissue contrast resolution compared to radiograph findings and will provide reconstructed images of the target area at different levels. CT scan is a practical method for diagnosing space‐occupying and deforming lesions and a suitable method for choosing surgical approaches. Therefore, to correctly interpret waste, it is necessary to know about its natural forms (Forrest, 2011).

## 2 MATERIALS AND METHODS

### 2.1 Collection of the specimens

In this study, 10 adult Rhesus monkeys (5 females and 5 males) with an average weight of 6.5 kg were used. The samples were prepared in the Laboratory Animals Department from the monkeys of Pardisan Park.

At first, the health status of the animals was confirmed by clinical examinations, and the absence of bone deformities that could indicate metabolic bone disease was noted. The monkeys were in relatively similar conditions in terms of food, storage temperature, and humidity. At the time of selection, attention was paid to the animal's diet, the intake of vitamins (especially vitamin D) and minerals (especially calcium and phosphorus) and the daily intake of sunlight. To prevent harm to the animals, after being bound, each one was transported to the small animal hospital of the Faculty of Veterinary Medicine of the University separately in special and well‐ventilated boxes.

### 2.2 Drugs and method of anaesthesia

To prepare radiographs and CT scans of the skulls of the mentioned monkeys, who were fasting, it was necessary to apply general anaesthesia to create the best imaging mode. Therefore, a combination of two drugs, ketamine (with a dose of 5 mg/kg) and xylazine (with a dose of 0.25 mg/kg), was injected intramuscularly into each monkey. After 5–10 min of induction of anaesthesia, each animal was placed in the expected state. During anaesthesia, sterile vitamin A ointment was used in the eyes of monkeys to prevent corneal dryness (Naccarato & Hunter, 1979).

### 2.3 Radiographic imaging

The following equipment was used to prepare radiographs of the monkeys’ heads:

- 24 × 18 size cassette.
- Automatic processor device.
- Stepped aluminium phantom: This phantom has 15 steps; the thickness of the first step is 0.5 mm, and the thickness of each step is increased by 0.25 mm, so the thickness of the last step is equal to 4 mm.
- Kodak, Carestream, DirectView, classic CR, Toshiba Dc‐12m computer radiography machine.

After the induction of anaesthesia, the animal was placed in the standard dorsal‐abdominal and lateral imaging positions with vertical radiation; after entering the information of each monkey on the page of the processor device, images with technical factors including 8 mAs and 60 kVp, and by placing a stepped aluminium phantom, it was prepared for each film.

To make a lateral projection, the head was placed in such a way that the nasal septum was parallel to the cassette. The beam was centred in the area between the ear and eye and behind the zygomatic bone and was completely limited by the collimator. In the dorsoventral view, after placing the animal on the sternum, a lead glove was placed on the neck and the back of the skull to ensure the correct position. In this case, the hard palate will be parallel to the cassette. The beam was irradiated centred on the region between the eyes and ears, right on the centre line, and was completely limited by the collimator. The obtained images were transferred to the image archiving and exchange system to store, download and view them. Bone structures of the skull were identified and labelled as much as possible, and their density was evaluated. A backup file was prepared on a compact disc to record and maintain improvements.

To compare the obtained bony data from radiographs with the CT number in CT scan images, the zygomatic bone, which consists of the zygomatic process of the temporal bone and the temporal process of the zygomatic bone, in the dorsoventral view, as the base structure for radiographic examinations and transverse sections, was considered in the CT scan studies.

All of the photographs were opened using the ImageJ software programme, and quantitative density checks were performed on them. The phantom image was considered the base density and was used to calibrate the software programme. In this way, the density of the background part of the photograph, equivalent to zero millimetres of aluminium, and the density of the other steps, equivalent to the thickness of the said step, was expressed in millimetres of aluminium, and the graph related to the calibration of each image was saved. Then, the studied area was selected on each side, and its average density was obtained based on millimetres of aluminium. Finally, the average density of the right and left zygomatic bones was calculated for all samples.

### 2.4 Computed tomographic imaging

The used device was a Somatom‐2 detectors machine manufactured by Siemens (third‐generation spiral with two rows of detectors) with 130 kVp and 77 mAs and 1 mm slice thickness. After preparing the radiographs under general anaesthesia conditions (continuous breathing and heart rate monitoring), the monkeys were placed one by one on the CT scan bed in the sternal position. The head was placed on the radiolucent foam, and both sides of the monkey's body were fixed using this foam. Then, the information about each animal, including the number assigned to each animal, gender and the date was recorded in the device. After applying the appropriate settings for the device, the range of the topogram was selected from the front of the nostrils to the end of the head. After this, cross‐sectional images of the entire length of the head were taken.

The images were transferred to the computer and reconstructed from the beginning to the end of the head by Singo software made by Siemens company, in two‐dimensional transverse and longitudinal and three‐dimensional bone formats, and the bony structures of the skull, sinuses, conchas, teeth and so on were identified and named, and their density was determined by checking the CT number. In the 3D reconstruction of the images, the osseous‐shaded‐VP pattern was used. The images were sent to the PACS system, and a backup file was prepared on a compact disc to record and maintain the corrections.

In the Singo software, to find the CT number, the images were loaded one by one in the volume programme. After adjusting the assessment limits of the zygomatic bone (between 700 and 3000 Hounsfield units), the border of the zygomatic bone was determined from the posterior part of the zygomatic process of the temporal bone to the anterior part of the zygomatic process in transverse sections. Due to the absence of a fibrous suture on both sides of the zygomatic bone, to have a correct understanding of the beginning and end of this part, each image was loaded in the 3D programme, and the exact location of the zygomatic arch was visualized with a 3D view and considered in the volume programme. Finally, after determining the limits of this bone, the evaluation was done, and the average and standard deviation of the bone density with Hounsfield units were recorded and kept for each sample.

### 2.5 Morphometry of the skull

**FIGURE 1:**
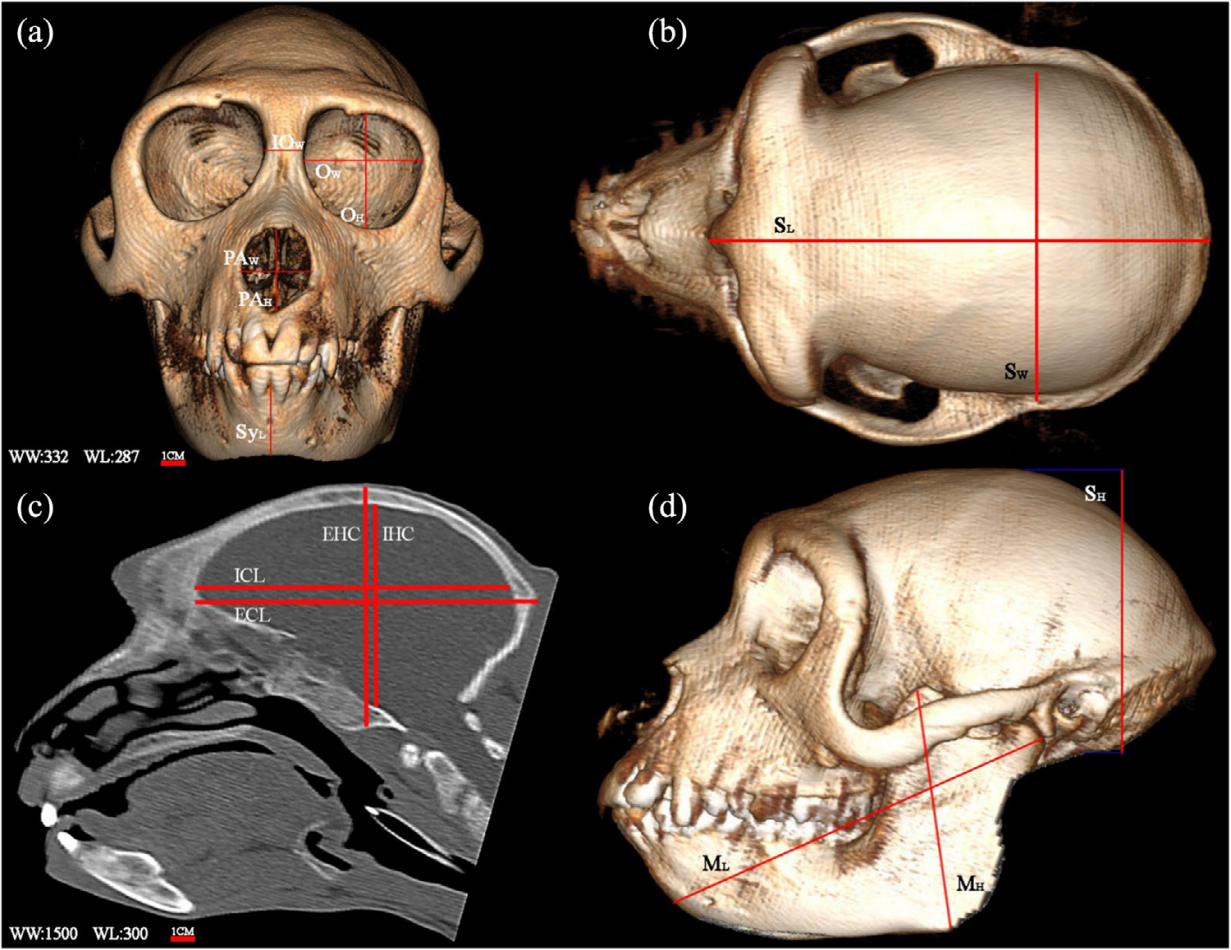
Morphometric parameters showed in skull: (a, b and d) 3D computed tomography (CT) scan of Rhesus skull. (c) 2D longitudinal CT scan of Rhesus skull which shows internal morphometric features: (IO*_L_*) inter orbital length; (O*_w_*) orbit width; (O*_H_*) orbit height; (PA*_W_*) piriform aperture width; (PA*_H_*) piriform aperture height; (Sy*_H_*) symphysis of mandible height; (S*_L_*) skull length; (S*_W_*) skull width; (IHC) internal height of cranium; (EHC) external height of cranium; (ILC) internal length of the cranium; (ELC) external length of the cranium; (S*_H_*) skull height; (M*_H_*) mandibular height; (M*_L_*) mandibular length.

Considering the special characteristics of this monkey species and its similarities to humans, in addition to examining the anatomical characteristics, we also examined the morphometric characteristics of the skull. The examined morphometric parameters and the descriptions of each one are available in Table 1 and are also shown in Figure 1.

**TABLE 1:** Morphometric result.

Parameters	Description	Parameters	Description
Skull length (SL)	Measured as distance between the rostral point of the incisive bone to the external occipital protuberance of the occipital bone	Mandibular length (M length)	Distance between the highest point of the mandibular condyle and lowest part of mandibular symphysis
Skull height (SH)	Measured as distance from the summit of the frontal bone to the tip of the para‐condylar process	Symphysis of mandibular length (Sy length)	Distance between the highest point of the mandibular symphysis and its lowest part
Skull width (SW)	The length between the most protruding points in the parietal and temporal bones on both sides of the skull	Cranial length (CL)	Distance between the nuchal crest and the cranial rims of the eyes
Inter Orbital width (IO width)	Measured as distance between two orbits	Cranial width (CW)	Distance between the most lateral points of the cranial cavity at the level of the external acoustic meatus
Foramen magnum length (Fm length)	Longitudinal distance between the margins of the foramen magnum	Facial length (FL)	Distance between the nasofrontal suture and the most rostral point of the incisive bone
Foramen magnum width (Fm width)	Transverse distance between the margins of the foramen magnum	Nasal length (NL)	The distance between the tip of the nose and the beginning of the cribriform plates
Piriform aperture height (PA height)	Longitudinal distance between the top and bottom of the nasal cavity	Internal height of cranium (IHC)	The highest height of the internal part of the cranium
Piriform aperture width (PA width)	Transverse distance between the lateral margins of the nasal cavity	External height of cranium (EHC)	The highest height of the external part of the cranium
Orbit height (O height)	The height of the eye ball	Internal length of the cranium (ILC)	The maximum length of the internal part of the cranium
Orbit width (O width)	The width of the eye ball	External length of the cranium (ELC)	The maximum length of the external part of the cranium
Mandibular height (M height)	Distance between the highest point of the mandibular coronoid and lowest part of mandibular body		

*Note*: Parameters and descriptions in the skull of Rhesus monkey.

### 2.6 Statistics

A statistical analysis was performed on the morphometric data in males and females using SPSS (Statistical Package for the Social Sciences) version 26 software. The test used to determine the significance of the difference between the morphometric characteristics of males and females was pair sample *t* test. *p*‐Value less than 0.05 was reported as a significant difference.

## 3 RESULTS

The radiographic images are named in Figures 2 and 3. The names of 2D CT scan images are also given in Figures 4, 5, 6, 7 and 9, and also 3D CT scan images are given in Figure 8. Moreover, the results of skull morphometric studies are presented in Tables 2 and 3.

**FIGURE 2:**
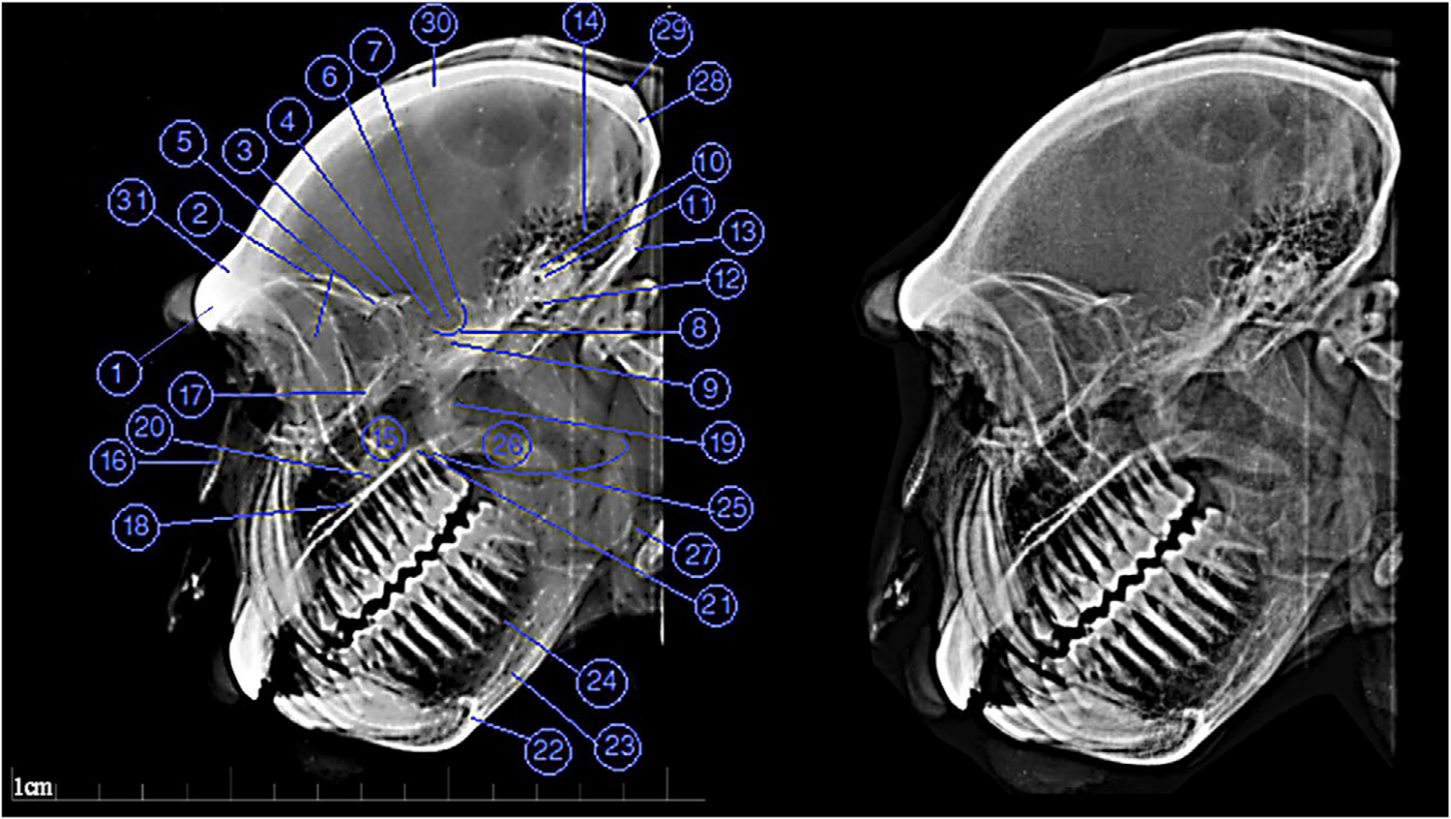
Lateral radiograph of Rhesus skull: 1, Frontal bone; 2, wall of orbital cavity; 3, wing of sphenoid; 4, rostral clinoid process; 5, ethmoidal bone; 6, hypophyseal (pituitary) fossa; 7, caudal clinoid process; 8, dorsum sellae; 9, body of sphenoid; 10, squamous part of temporal; 11, internal ear foramen; 12, external ear foramen; 13, mastoid process of temporal bone; 14, mastoid air cells; 15, maxillary sinus; 16, nasal bone; 17, floor of orbital cavity; 18, hard plate; 19, pterygoid process of sphenoid; 20, zygomatic process of maxilla; 21, palatine bone; 22, mental foramen; 23, body of mandible; 24, mandibular canal; 25, ramous of mandible; 26, soft plate; 27, angle of mandible; 28, occipital bone; 29, external occipital protuberance; 30, parietal bone; 31, frontal bone.

**FIGURE 3:**
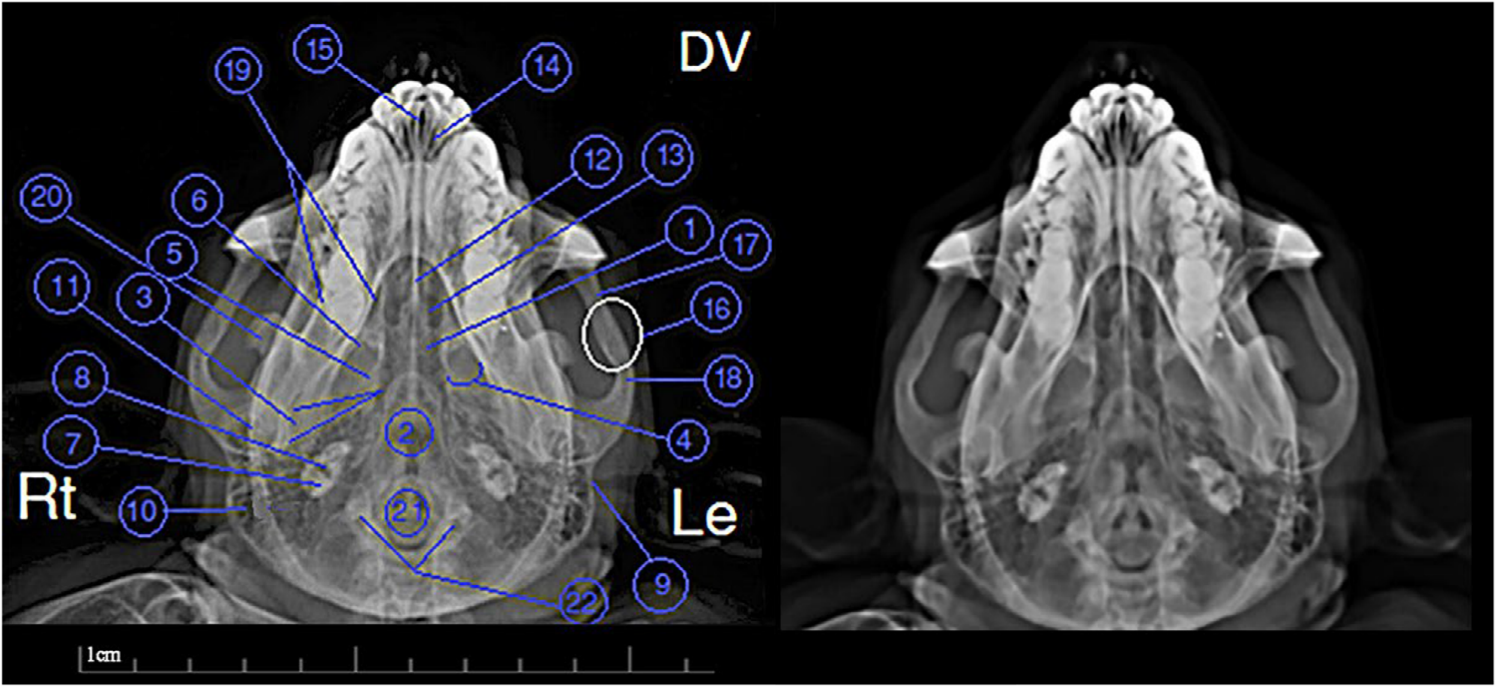
Dorsoventral radiograph of Rhesus skull: 1, sphenoid bone; 2, occipital bone; 3, styloid of temporal bone; 4, pterygoid process of sphenoid; 5, medial plate of pterygoid; 6, external plate of pterygoid; 7, squamous part of temporal; 8, internal foramen of carotid canal; 9, external acoustic meatus; 10, mastoid process of temporal; 11, condylar process of mandible; 12, vomer; 13, choana; 14, maxillary bone; 15, incisive canal; 16, suture of temporal and zygomatic bone; 17, temporal process of zygomatic bone; 18, zygomatic process of temporal bone; 19, sides of mandible; 20, mandible; 21, foramen magnum; 22, occipital condyle.

**FIGURE 4:**
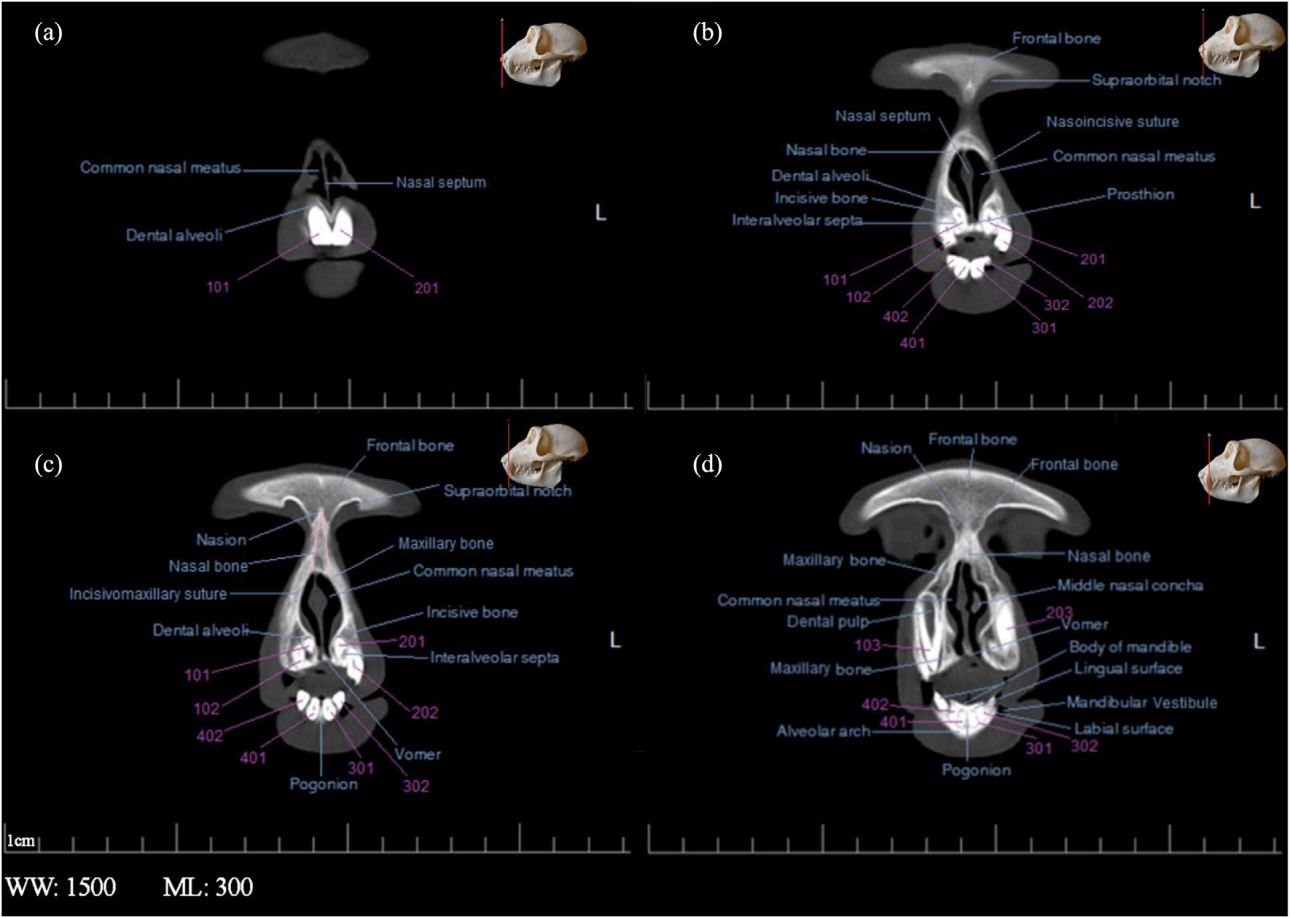
2D transverse computed tomography (CT) scan of the skull of Rhesus monkey: (a) at level of first maxillary incisive teeth; (b) at level of second and third maxillary incisive teeth; (c) a little later than (a); (d) at level of maxillary canines and mandibular incisors.

**FIGURE 5:**
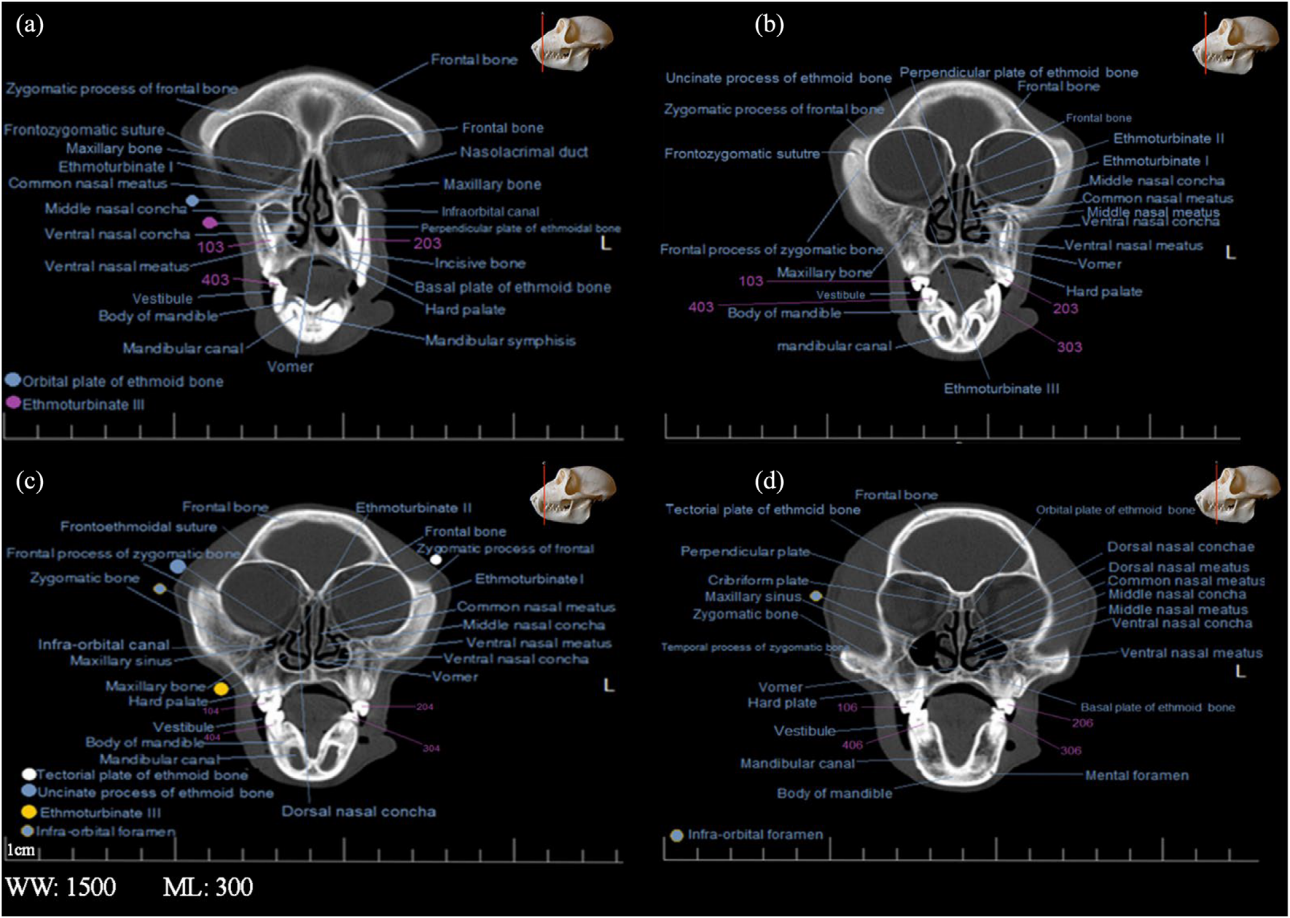
2D transverse computed tomography (CT) scan of the skull of Rhesus monkey: (A) at the level of maxilla and mandible canine teeth; (B) a little later than (A); (C) at the level of first pre‐molar teeth; (D) at level of first molar teeth.

**FIGURE 6:**
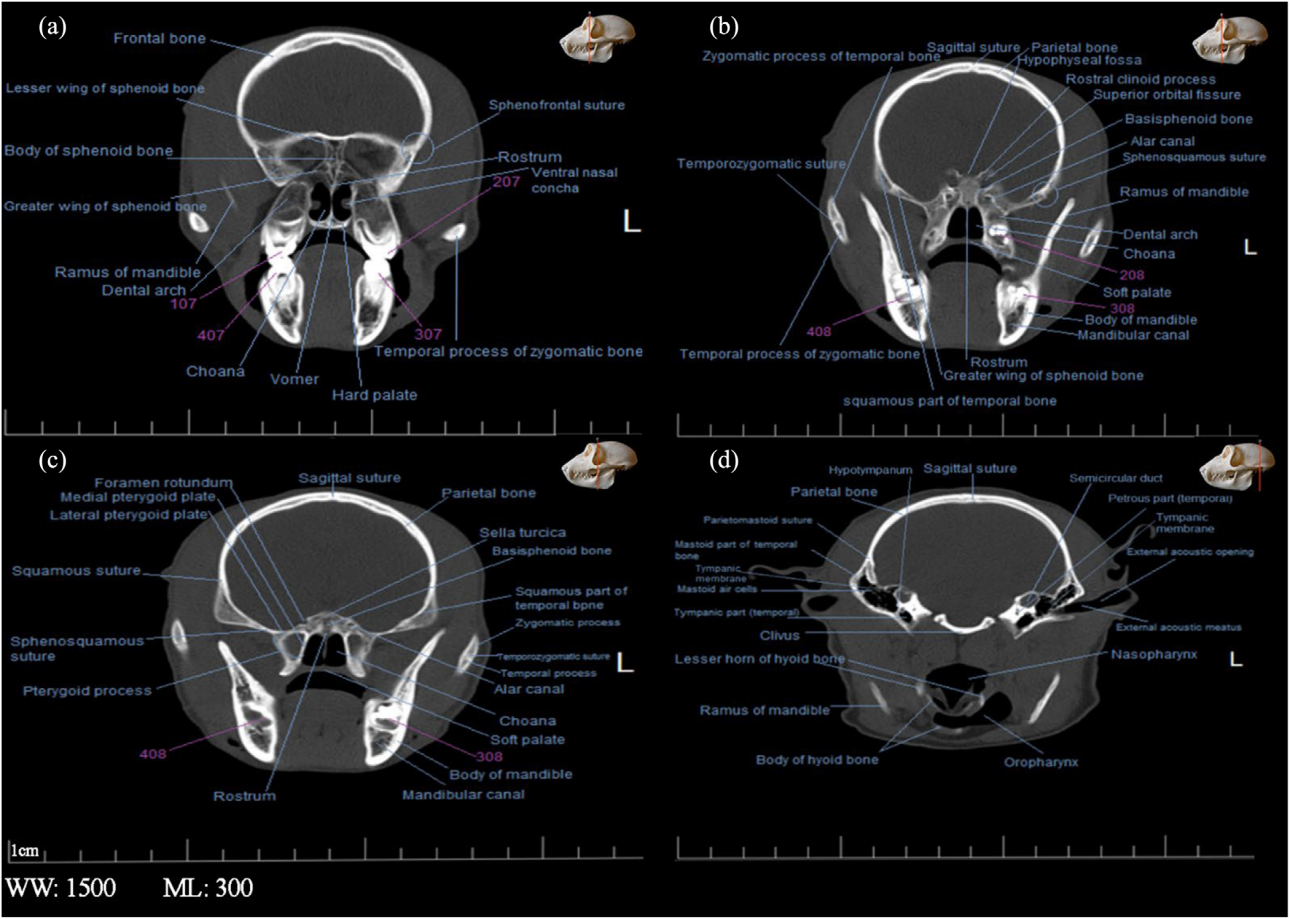
2D transverse computed tomography (CT) scan of the skull of Rhesus monkey: (A) at level of second molar teeth; (B) at level of third molar teeth; (C) at level of third mandibular molar teeth; (D) at level of the hyoid, inner and middle ear.

**FIGURE 7:**
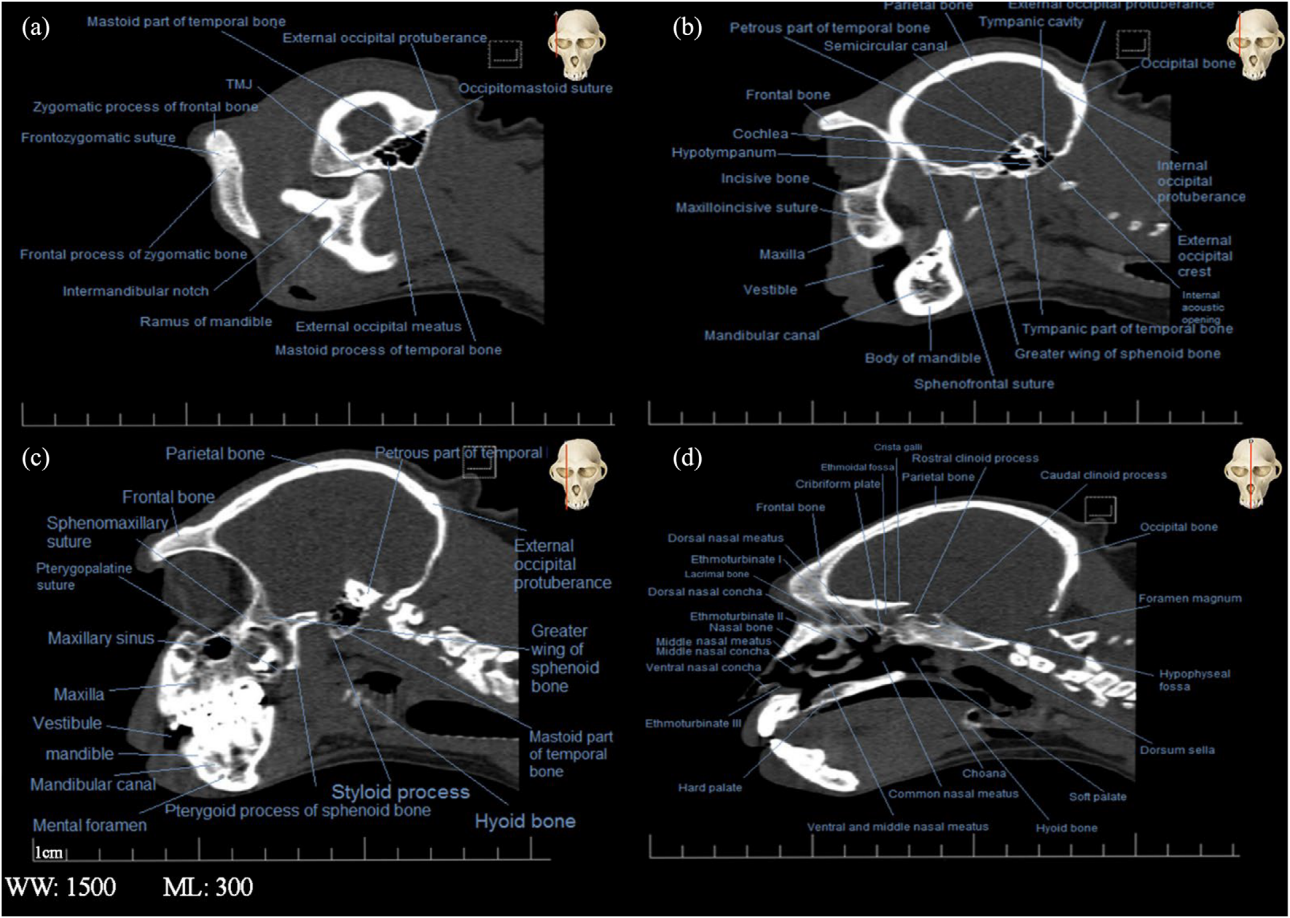
2D longitudinal computed tomography (CT) scan of the skull of Rhesus monkey: (A) at level of external occipital meatus; (B) at level of angle of mandible; (C) at level of styloid process of mandible; (D) median section.

**FIGURE 8:**
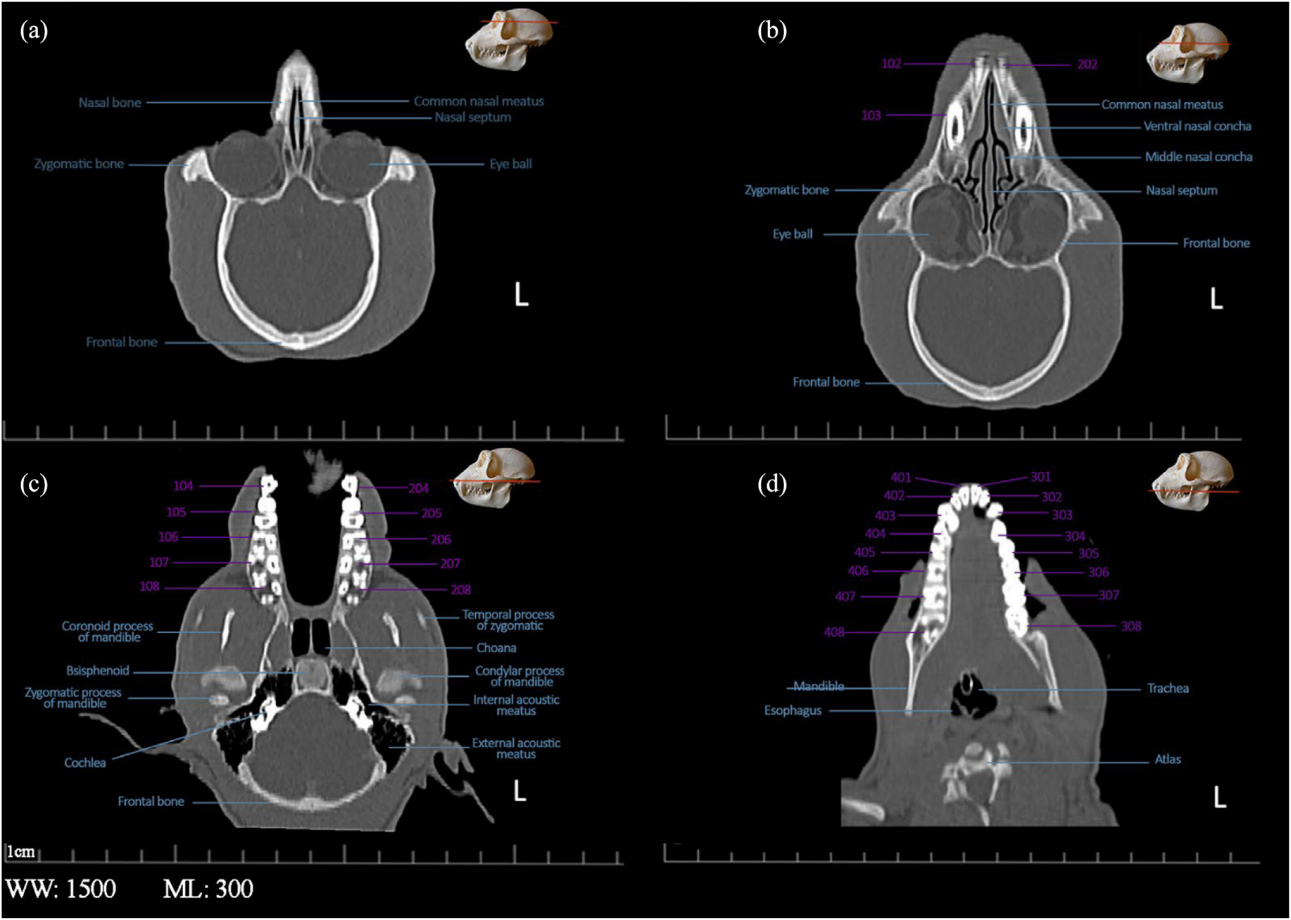
2D coronal computed tomography (CT) scan of the skull of Rhesus monkey: (A) at level of mid part of eye; (B) at level of nasal cavity; (C) at level of temporomandibular joint; (D) at level of mandibular tooth.

**TABLE 2:** Number of roots per tooth in Rhesus monkey.

	I1	I2	C	PM1	PM2	M1	M2	M3
Maxilla	1	1	1	3	3	3	3	3
Mandible	1	1	1	2	2	3	3	3

*Note*: I, incisor tooth; C, canine tooth; PM, pre‐molar tooth; M, molar tooth.

**TABLE 3:** External morphometric features of Rhesus monkey skull.

	S length	S width	S height	IO width	Fm length	Fm width	PA height	PA width	O height	O width	M height	M length	Sy height	C length	C width	F length	N length
Male (mean)	10.84	7.44	5.84	0.84	1.54	1.35	2.60	1.41	2.39	2.62	4.84	7.72	1.81	9.06	5.96	2.70	1.32
Female (mean)	9.06	6.01	5.24	0.62	1.29	1.21	1.82	1.16	2.08	2.40	4.59	7.59	1.34	8.62	6.07	1.82	1.28
Mean ± SD (F&M)	9.95 ± 0.89	6.73 ± 0.71	5.54 ± 0.31	0.73 ± 0.12	1.41 ± 0.15	1.28 ± 0.08	2.21 ± 0.39	1.29 ± 0.13	2.24 ± 0.20	2.51 ± 0.15	4.72 ± 0.13	7.65 ± 0.10	1.58 ± 0.26	8.84 ± 0.23	6.02 ± 0.17	2.26 ± 0.47	1.30 ± 0.18

*Note*: (S) skull, (IO) inter orbital, (Fm) foramen magnum, (PA) piriform aperture, (O) orbit, (M) mandible, (Sy) symphysis of mandible, (C) cranium, (F) frontal, and (N) nasal. Orbit and mandibular morphometry have measured as average of left and right. The unit of measurement is centimetre.

### 3.1 Radiography

Figures 2 and 3 are lateral and dorsoventral radiographs of the head of a male monkey, respectively. It should be noted that there was no significant difference between males and females. Therefore, due to the larger size of the skull of the male monkey, figures related to this genus have been used to better display the structures.

The frontal bone was seen as two parallel radiopaque lines coming forward and downward. The outer radiopaque line represented the outer membranous plate of the frontal bone, and the inner line represented the inner membranous plate. These two parallel lines were separated in the forehead area (Figure 2/1). The frontal sinus in the Rhesus monkey was not visible in both lateral and dorsoventral views, which could indicate the degeneration of this sinus in this species.

The anterior surface of the sphenoid bone (Figure 2/9) was located behind the cribriform plate of ethmoidal bone (Figure 2/5) and under the small wing of the sphenoid bone. In the posterior part of the body of this bone, the shadow of the saddle area was seen, which had an oval structure. In the centre of this region, a brief radiolucency was observed, which indicated the shadow of the pituitary cavity (Figure 2/6). In the centre of the body of the sphenoid bone, which is a common region for observing the sphenoid sinus, there was no hypoattenuating area that could be named sphenoid sinus. In front of the centre of the body of this bone, a radiopaque curve was noticed, which indicated the inner surface of the large wing of the sphenoid bone (Figure 2/3). In the dorsoventral view, the body of this bone (Figure 3/1) was located in a pair between the palatine bone and the back of the head, and its opacity was less than the surrounding bones.

The squamous part of the temporal bone was observed as a circular radiopaque shadow in the lateral view (Figure 2/10) and an oval shape (Figure 3/7) in the dorsoventral view. The internal ear foramen (Figure 2/11) was seen in the lateral view. The external ear foramen in the side view (Figure 2/12) was observed below and in front of the internal ear foramen. This is while the internal and external ear foramen was not seen in the dorsoventral view.

In the lateral view, in the lower part of the temporal bone, the mastoid process (Figure 2/13) was seen as a prominent radiopaque area. In the same view, behind the spinous part of the temporal bone to the mastoid process of the temporal bone, many radiolucent points were visible, which indicated the mastoid air cells (Figure 2/14). These points were also observed in the dorsoventral view, in the mastoid part of the temporal bone (Figure 3/10). In the lateral view, in front of the anterior surface of the first cervical vertebra (atlas), below the spinous part and behind the condylar process of the mandible (Figure 2), a fine radiopaque protrusion was observed, which is the styloid of the temporal bone (Figure 2). The foramen of the carotid canal in the dorsoventral view (Figure 3/8) was located as a relatively oval radiolucent shadow in the centre of the spinous part. This foramen is where the internal carotid artery passes.

In the lateral view, the only visible portion of the ethmoid bone was the ethmoidal labyrinth (Figure 2/5), which was perceived as a radiopaque and porous area with a grey shade. Generally, this section reaches the lacrimal bone in front. Below this area, a radiopaque line was seen up to the nasal bone, which indicated the upper wall of the maxillary sinus. The posterior region of the ethmoidal bone was defined by the anterior surface of the body of the sphenoid bone (Figure 2). In the maxillary sinus region, there were also grey shadows caused by overlapping dorsal, middle and ventral conchas in this area. This bone was not visible in the dorsoventral view (Figure 8).

In the lateral view, the nasal bone (Figure 2/16) was seen as a triangular radiopaque area with fine trabeculation. The tip of this triangle was downward. This bone was cartilaginous in front and was observed as a radiolucent area. Under the nasal septum, the alveolar process (Figure 2) was extended as a curved radiopaque line to the incisors.

Inside the maxillary bone was the maxillary sinus (Figure 2/15), which was seen as a large radiolucent area surrounded by radiopaque lines. The maxillary sinus starts from the inner edge of the first molar tooth and continues parallel to the hard palate to the root of the third molar tooth. Meanwhile, there is a thin bony wall between the maxillary sinus and the roots of the first and second molar tooth. In the lateral view, the right and left sinuses overlapped. In the dorsoventral view, this part was not visible due to the superimposed bony structures that covered the sinus. In the middle of the maxillary sinus, the zygomatic process of the maxillary bone (Figure 2/20) was seen as a radiopaque shadow on top of the hard palate, with the body of the zygomatic bone (Figure 2) above as two radiopaque lines. It was seen parallel, extending.

The hard palate was seen as two parallel radiopaque lines in the lateral view. The upper line indicated the floor of the nasal cavity, and the lower line indicated the roof of the oral cavity. It should be noted that the soft palate was placed along the hard palate (Figure 2/26). In the anterior third, the incisor or intermaxillary bone was seen, which was clearly visible in the dorsoventral view (Figure 3/14), but not in the lateral view due to the dental structures. In the middle of this bone, the incisor canal (Figure 3/15) was observed as a radiolucent line.

In the dorsoventral view, the palatine bone was not visible due to the superimposition of the horizontal part of the mandible bone and the palatine bone. The back of the palatine bone was bounded by the sphenoid bone. In the lateral view, the horizontal part of the palatine bone was located as a radiopaque area in the roof of the oral cavity (Figure 2/21).

The frontal process of the zygomatic bone (Figure 2) was seen as two radiopaque lines. The anterior line, which was curved, indicated the anterior border of the lateral wall of the eye ball, and the posterior line came down from the upper border of the ethmoidal bone and reached the posterior border of the zygomatic process of the maxillary bone (Figure 2/20). The body of this bone (Figure 2) above the shadow of the hard palate was connected to the zygomatic process of the temporal bone in the back as a radiopaque area. In the dorsoventral view, the zygomatic bone, which consisted of the temporal process of the zygomatic bone in front and the process of the temporal bone in the back, was seen as a curved radiopaque area on both sides of the skull (Figures 3/17,18).

In the lateral view, behind the root of the canine tooth, a radiolucent circular foramen was observed, which indicated the mental foramen (Figure 2/22). The body of the mandible (Figure 2/23) extended posteriorly in a very radiopaque line from under the incisors to the angle of the mandible. Between the pre‐molar and molar teeth and the horizontal branch of the lower jaw, a radiolucent area was observed, which was the shadow of the mandibular canal (Figure 2/24). The vertical ramous of the mandible (Figure 2/25) extended from the angle of the mandible (Figure 2/27) to the occipital bone with less opacity than the horizontal branch. In the dorsoventral view, the body and ramous of the mandible were lying on the superimposed bones; however, the border of the bone body was visible in the image as a radiopaque area (Figure 3/19).

In the lateral view, the occipital bone was located in a very radiopaque line between the parietal bone above and the temporal bone at the base of the skull (Figure 2/28). Fibrous joints separating these bones were not visible in this view. A radiopaque ridge was seen in this bone, indicating the external occipital protuberance (Figure 2/29). On top of this projection was the scaly part of the occipital bone.

The parietal bone was placed in a very radiopaque line between the frontal bone in the front and the occipital bone in the back (Figure 2/30). In the dorsoventral view, it could not be seen due to the overlapping of this bone with its adjacent bones.

The lacrimal bone was seen as a radiopaque area behind the nasal bone and in front of the parotid bone and the frontal process of the zygomatic bone (Figure 2).

In the lateral view, the incisors of each jaw could not be seen separately. The anterior border of the maxillary incisors was located in front of the anterior border of the mandibular incisors. In the dorsoventral view, the upper incisors were in front of the lower incisors and could be seen separately. The roots of the maxillary canine teeth were located in the area that was in front of the nasal bone and above, under the floor of the eye ball. The roots of the canine teeth of the lower jaw were in the body of the mandible bone and ended in the mental foramen. These teeth were very big compared to other teeth and their anatomical parts could be seen well. Pre‐molar and molar teeth continued to about the end of the hard palate above and the body of the mandible below. The roots of the teeth could not be distinguished well in the radiograph.

### 3.2 2D CT scan

Sections of the transverse and longitudinal 2D CT scan were examined from the beginning to the end of the head, and all bony structures are named and described in Figures 3, 4, 5, 6. It should be noted that the description of anatomical regions is repeated in each image.

In the first section of the study, which is at the level of the first maxillary incisive teeth, the anterior part of the nasal septum is observed, that has less bone tissue, which indicated that the septum was cartilaginous in its beginning (Figure 4A). In the next section, the frontal bone structure was seen as a triangle, which had radiolucent curved depressions called supraorbital notches at its lateral vertices (Figure 4B). A little further back at the same level, a radiopaque point called Nazion could be seen in the centre of the frontal–nasal–maxillary suture (Figure 4C). At this point, the nasal bone was observed as radiopaque, similar to the triangular area under this point. In the lower part of the nasal cavity, between the two maxillary first incisors, there was a radiopaque point that was the beginning of the vomer bone. Moreover, between the first two incisors of the mandible, there was a radiopaque area on the alveolar arch, which is called pogonion. In the maxillary canines and mandibular incisors section, the dental pulp in the radiolucent area was covered by surrounding radiopaque structures (enamel and cementum), which was true in all teeth in other sections. A part of the middle turbinate on the left side was on the upper half of the nasal septum (Figure 4D).

The nasolacrimal canal opens from the lacrimal bone towards the nasal bone, in Figure 5A, it was seen as a radiolucent spot on the maxilla bone on the left side. Both nasolacrimal ducts were seen at their junction with the lacrimal bone. The left duct was a radiolucent circle, and the right duct was more radiopaque than the opposite duct, which indicated that the structure was full. In the next section, which is at the level of the maxillary and mandibular canine teeth and a little more posterior than the previous section, the frontal appendage of the zygomatic bone was seen as a radiopaque area on the side and towards the bottom of the eye ball (Figure 5B,C). The next section is at the level of the first molar teeth. At this point, the ethmoidal turbinate of the ethmoidal bone was enclosed by the ceiling membrane above, the orbital cavity on the sides and the dorsal nasal concha below. The maxillary sinuses were observed in the maxillary bone on both sides as radiolucent areas. In the body of the mandible bone, the mental foramen was seen as a radiolucent circle (Figure 5D).

In the section of secondary molar teeth, the small wings of the sphenoid bone at the top and its big wings at the bottom of the body of this bone were seen as relatively radiopaque. On the ventral surface of this bone, a triangular radiopaque shadow was observed, the top of which reached the top of the vomer bone at a radiolucent point (Figure 6A). In Figure 5B, the right and left parietal bones meet at the back of the skull by a longitudinal suture. Moreover, the squamous part of the temporal bones and the large wing of the sphenoid were connected in the sphenoid–squamous joint. In the next section, which is at the level of the end of the maxillary dental arch, in the continuation of the alar canal, a round foramen was observed that opened to the middle skull cavity. It should be noted that this channel originates from the middle cranial cavity and goes to the mandibular–palatal cavity, which contains the maxillary nerve (Figure 6C). In Figure 6D, the eardrum could be seen. In the continuation of the external ear canal, the external ear foramen was seen.

In image A of Figure 7, which shows the longitudinal CT scan section of the Rhesus monkey skull, the external occipital meatus was seen as a radiopaque area above the mastoid part of the temporal bone. The occipital bone was articulated with the temporal bone by the occipito‐mastoid suture. In Figure 7B, different parts of the inner ear were seen. In Figure 7C, parts of the hyoid bone were seen. In Figure 7D, the mesh plate was seen as a relatively radiopaque porous diagonal area at the bottom of the radiolucent ethmoidal cavity.

### 3.3 3D CT scan

In the 3D reconstruction of the Rhesus monkey skull, using the bone model and after removing the skin, muscle structures, vessels and nerves from the skull by Singo software, to facilitate the expansion of bone components, the images were saved in four views and then named in Figure 8.

### 3.4 Teeth

**FIGURE 9:**
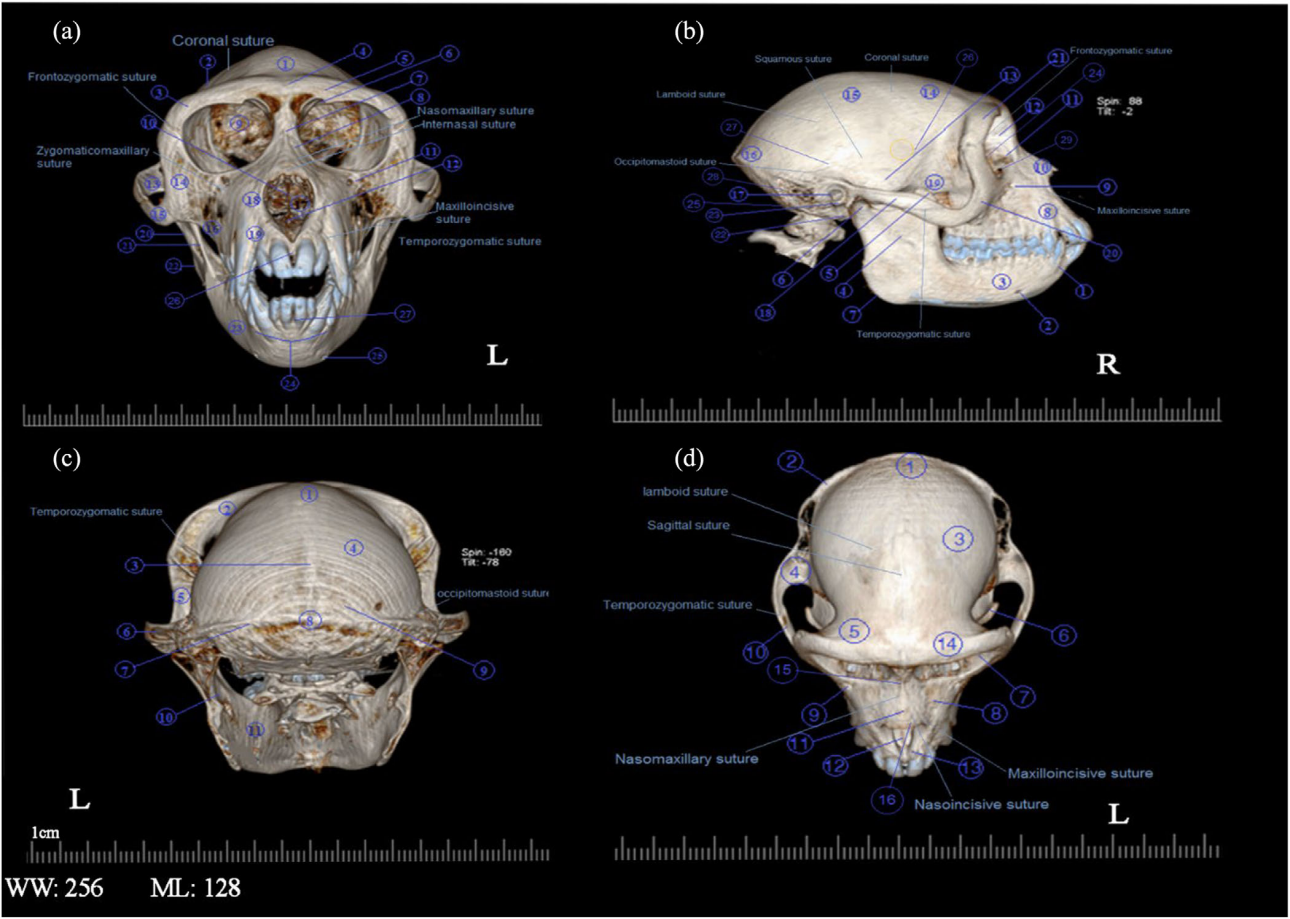
3D computed tomography (CT) scan of the skull of Rhesus monkey: (A) anterior view; (B) lateral view; (C) caudal view; (D) dorsal view.

**FIGURE 10:**
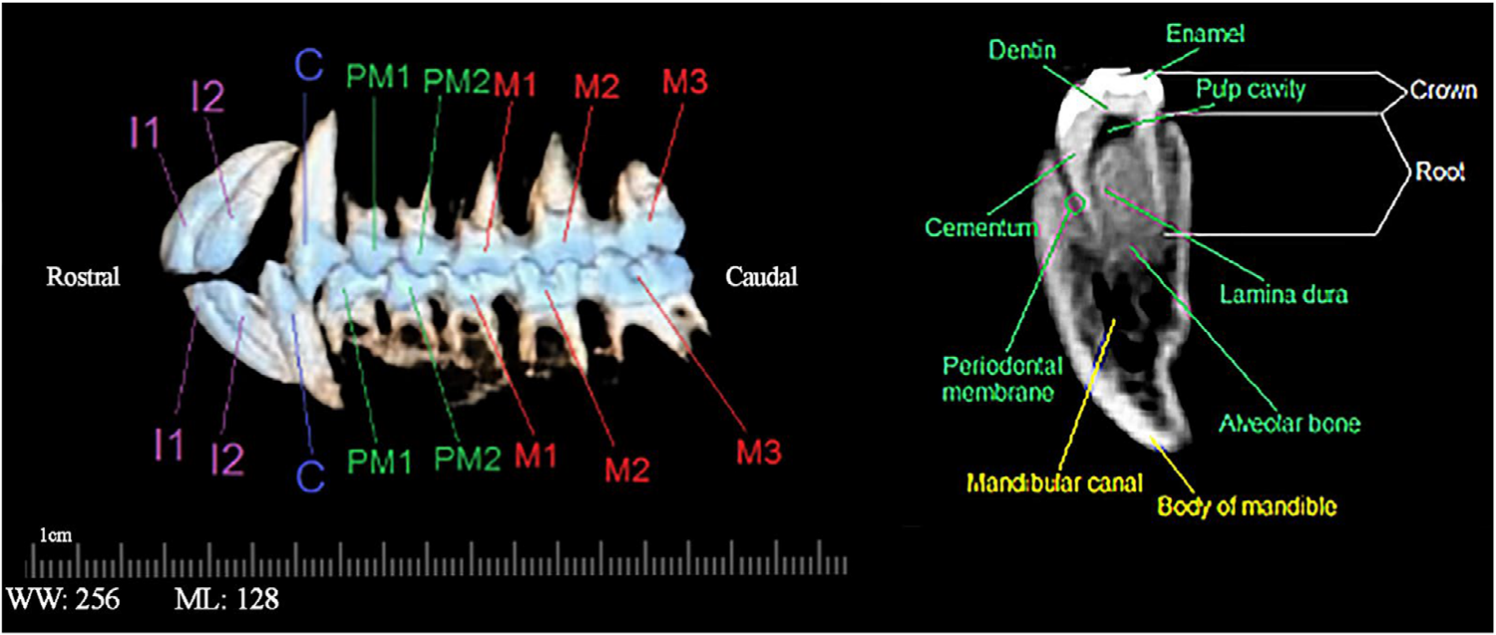
Dental arrangement of Rhesus monkey in 3D reconstruction using bone model along with different parts of a tooth in the mandible.

The number of teeth in an adult monkey was 32; molar teeth had a bilophodont arrangement. In such a way that each tooth had a transverse depression in its middle (Figures 9 and 10). The number of roots of each tooth was counted and recorded, in both the transverse 2D reconstruction and in the 3D reconstruction (Table 2). The number of roots of the first and second incisor and canine teeth was 1, and all the premolar and molar teeth of the maxilla had three roots. The number of roots in the premolar and first molar teeth of the mandible was 2, and the second and third molar teeth had three roots. You can see the structure of a mandibular tooth in Figure 8. The formula for the permanent teeth of the Rhesus monkey is as follows:

$$2\times I\frac{2}{2}C\;\frac{1}{1}PM\frac{2}{2}M\frac{3}{3}=32$$

### 3.5 Morphometry

The results of morphometric characteristics can be seen in Tables 3 and 4. According to the measurements, the length (SL), width (SW) and height (SH) of the skull were 9.95 ± 0.89, 6.73 ± 0.71 and 5.54 ± 0.31 cm, respectively, in males and females. Moreover, the height of the inner (IHC) and outer (EHC) parts of the skull was calculated to be 4.31 ± 0.18 and 5.38 ± 0.25 cm on average in males and females, respectively. Based on the results of pair sample *t* test, the size of SL, SW, SH, inter orbital length, piriform aperture height, piriform aperture width, mandibular height, symphysis of the mandible, cranium length, facial length and external length of the cranium in males and females had a significant difference (*p*‐value ≤0.05).

**TABLE 4:** Internal morphometric features of Rhesus monkey skull.

	IHC	EHC	ICL	ECL
Male (mean)	4.34	5.58	7.20	8.85
Female (mean)	4.27	5.18	7.11	8.09
Mean ± SD (F&M)	4.31 ± 0.18	5.38 ± 0.25	7.16 ± 0.13	8.47 ± 0.39

*Note*: The unit of measurement is centimetre.

Abbreviations: ECL, external length of the cranium; EHC, external height of cranium; ICL, internal length of the cranium; IHC, Internal height of cranium.

## 4 DISCUSSION

In the LeMay study, 10 examples of asymmetry in the brain and skull of non‐human primates were investigated. According to this study, structures, such as frontal lobes, brain sulcus, temporoparietal region and lateral brain sulcus, have asymmetry (LeMay et al., 1982). In this study, no asymmetry was observed in the temporal and temporal regions in any of the sections.

In another investigation, a biopsy was performed on the brain of a Rhesus monkey using a robot equipped with a CT scanner to identify the tumour. Once the tumour was detected by CT, a simple guiding device guided the robot towards the tumour, and sampling was performed. This method was described to be much faster than manual methods (Flügel & Rohen, 1991).

The ratios of anterior facial and larynx bones in monkeys and humans at different ages were investigated based on CT scans and radiography in another study (Flügel & Rohen, 1991).

The evolutionary process of the formation of skull bones and facial nerves in humans and monkeys, as well as the morphometric study of teeth and jaws with CT and radiographs, was conducted in another study. Parameters, such as prognathic angle, clivus angle, palate‐incisive angle and interincisive angle, were measured in monkeys and humans and all parameters were somewhat similar in infants. This was while certain differences were observed in the growth pattern of monkeys and humans. In the monkey, significant growth of the prognathic angle was observed, whereas the growth of the neurocranium was done slowly. In monkeys, the flattening of the base of the skull is observed, whereas in humans, the change in proportions is not noticeable compared to infancy (Flügel et al., 1993). The flattening of the base of the skull was seen in the radiographs and CT scans performed in this study.

In the Losken study, three general shapes of the nasal septum in primates were classified, according to which the chimpanzee, Rhesus and baboon have a triangular septum (Losken et al., 1994). The triangular shape of the nasal septum in the radiographic and CT scan images confirms this study. This triangular blade was articulated to the frontal bone by the radiolucent vertical forehead–nasal suture in the lateral images obtained from the radiography above (Figure 2).

The ratio of external cortical bone variables in the skull of Rhesus monkeys was investigated using the ultrasonic method in another study. In this study, 28 cylindrical regions were isolated from all 6 bone samples of adult Rhesus monkeys. Thickness, density and a set of ultrasound wave propagation speeds were measured in each sample to calculate the ratio of variables in three dimensions. The results showed that there are significant differences in the ratio of variables in different areas of the Rhesus skull. In this study, it was found that the cortical bones of the Rhesus monkey skull can be a model similar to some parts such as the supraorbital region. The differences with the human skull are said to be due to differences in the proportions of cortical material (Wang & Dechow, 2006). The difference in the thickness of different parts of the skull in this study was also visible with the change in opacity. In such a way that, in areas such as the body of the mandible bone, some bones of the calvaria, and the floor of the skull cavity in radiography and CT scan, and areas such as the ossicles of the middle ear in CT scan, the bone density was much higher than in areas such as the cribriform plate or sphenoid bone.

In 2006, a study was conducted on the closing pattern of 28 cranial sutures in Rhesus monkeys of a specific age and sex. According to this research, the majority of animals die before most of the cranial sutures close. A significant difference was observed in the closing time of each suture in male and female Rhesus monkeys. The age limit in which the most fusion takes place is between the ages of 5 and 15 years. The sutures are closed less in the face than in the skull. Until puberty, the closure of the sutures, especially the facial sutures, is more frequent in males than in females, which can explain the sexual dimorphism in the Rhesus skull (Wang & Dechow, 2006).

A study was conducted on the anatomical atlas of the Rhesus monkey brain based on the functional near‐infrared spectroscopy imaging method. In this method, brain activity is examined by hemodynamic responses related to neural behaviours. Numerical simulation, hypothetical experiments and animal studies showed that this method is one of the advanced calculations in the construction and processing of 3D image findings (Xu et al., 2012).

A year after this research, anatomical and radiological results of cochlear ear transplantation in seven Rhesus monkeys were examined by CT scan and cone beam CT by another group. Several comparative measurements were made with 10 human temporal bone samples to investigate the similarities and differences between human and Rhesus monkey inner ears. This research found that the cochlear transplant process in monkeys is very similar to humans. However, there is a significant difference in the angle of the external ear canal of humans and monkeys (Marx et al., 2013).

In Rhesus monkey, unlike other species, such as Ile de France sheep, Alborz wild sheep, Saanen goat and also human, only the maxillary sinus was observed (Kawarai et al., 1999; Masoudifard et al., 2022; Tohidifar et al., 2020; Zehtabvar et al., 2022).

Table 5, which is the morphometric data of the skull in different species of animals, is obtained from the studies conducted on the skull of different animals. Considering the multitude of morphometric features reported in each article, only a few of these features that had a single definition in all articles were collected and finally, in order to compare the species as well as possible, the ratio of the length of the cranium to skull (CL/SL) and also the length of the nasal cavity to the skull (NL/SL), were calculated.

**TABLE 5:** Comparison of morphometric features and ratios in the skull of Rhesus monkey with other animals.

	S length	C length	N length	C width	CL/SL	NL/SL
Rhesus monkey	9.95 ± 0.89	8.84 ± 0.23	1.30 ± 0.18	6.02 ± 0.17	0.88	0.13
Round‐shaped skulls, feline (Künzel et al., 2003)	8.44 ± 0.67	3.64 ± 0.38	3.36 ± 0.27	4.41 ± 0.16	0.43	0.39
Triangular skulls, feline (Künzel et al., 2003)	9.77 ± 0.58	4.48 ± 0.25	3.82 ± 0.26	4.41 ± 1.53	0.45	0.39
Cuneiform skulls, feline (Künzel et al., 2003)	9.39 ± 0.68	4.27 ± 0.35	3.73 ± 0.25	4.23 ± 0.14	0.45	0.39
Shorthair cat (Ramos et al., 2021)	8.98 ± 2.98	8.23 ± 3.52	0.75 ± 0.21	4.31 ± 0.59	0.91	0.08
Dunkey (Zhu et al., 2014)	44.30 ± 5.35	20.782 ± 2.2	20.73 ± 3.50	9.08 ± 0.94	0.47	0.46
Ile de France sheep (Masoudifard et al., 2022)	25.3 ± 1.02	10 ± 0.66	Not reported	12.3 ± 0.91	0.39	Not reported
Alborz wild sheep (Zehtabvar et al., 2022)	25.28 ± 0.99	Not reported	15.02 ± 0.74	Not reported	Not reported	0.59
Saanen goat (Wang et al., 2021)	23.19 ± 2.36	8.37 ± 0.99	Not reported	11.54 ± 0.29	0.36	Not reported

*Note*: The unit of measurement is centimetre.

The CL/SL in the Rhesus monkey was 0.88, which was higher than most animals such as small ruminants and donkeys (Zhu et al., 2014), but compared to the Shorthair cat (Ramos et al., 2021), the cranium formed a smaller amount of the skull. Among the available reports, the lowest CL/SL was in the Saanen goat species (Wang et al., 2021). The NL/SL was calculated to be 0.13 in the Rhesus monkey, which was one third compared to the cat (Künzel et al., 2003).

The anatomical similarity between monkeys and humans encouraged us to compare the morphometric characteristics of these two species. Table 6, which is from the collection of articles on human morphometry and craniometry, has been compiled in order to compare with the morphometric data of the existing article. The comparison between the morphometric features of the human and Rhesus monkey skull indicated that all the characteristics were larger in humans, which is confirmed by the small head of the Rhesus monkey compared to humans.

**TABLE 6:** Comparison of morphometric features and ratios in the skull of Rhesus monkey with human.

	S length (Ulcay & Kamaşak, 2021)	S width (Ulcay & Kamaşak, 2021)	S height (Ulcay & Kamaşak, 2021)	IO width (Ulcay & Kamaşak, 2021)	Fm length (Singh & Talwar, 2013)	Fm width (Singh & Talwar, 2013)	PA height (Asghar et al., 2016)	PA width (Asghar et al., 2016)	O height (Kanjani et al., 2019)	O width (Kanjani et al., 2019)	M height (Direk et al., 2018)	M length (Direk et al., 2018)	Sy height (Direk et al., 2018)
Human	16.24 ± 0.62	12.94 ± 0.49	10.97 ± 0.4	2.26 ± 0.22	3.29 ± 0.14	2.74 ± 0.82	3.06 ± 0.34	2.41 ± 0.18	2.98 ± 0.17	3.18 ± 0.18	5.97 ± 0.53	12.09 ± 0.58	2.86 ± 0.48
Rhesus monkey	9.95 ± 0.89	6.73 ± 0.71	5.54 ± 0.31	0.73 ± 0.12	1.41 ± 0.15	1.28 ± 0.08	2.21 ± 0.39	1.29 ± 0.13	2.24 ± 0.20	2.51 ± 0.15	4.72 ± 0.13	7.65 ± 0.10	1.58 ± 0.26

*Note*: (S) skull, (IO) inter orbital, (Fm) foramen magnum, (PA) piriform aperture, (O) orbit, (M) mandible and (Sy) symphysis of mandible. The information of each parameter is an average of the left and right sides of the skull in male and female. The unit of measurement is centimetre.

The length, width and height of the skull in humans are reported to be 16.83, 14.06 and 10.97 cm, respectively (Ulcay & Kamaşak, 2021). Moreover, the width of the foramen magnum is 2.74 cm (Singh & Talwar, 2013). The height and width of the piriform aperture in humans are reported to be 3.03 and 2.38 cm, respectively, which are larger compared to the Rhesus monkey (Asghar et al., 2016). Moreover, the examination of the morphometric characteristics of the mandible also indicates a clear difference between the size of that in humans and monkeys (Direk et al., 2018). However, the comparison between the size of the eye ball in these two species, unlike other measured parameters, did not differ much, and this indicates that the volume ratio of the eye ball to the whole skull in Rhesus monkey is higher than that of humans (Kanjani et al., 2019).

## AUTHOR CONTRIBUTIONS

*Funding acquisition, validation, writing – review and editing*: Ali Reza Vajhi. *Funding acquisition, writing – review and editing*: Sarang Soroori. *Conceptualization, data curation, software*: Reihaneh Soflaei. *Methodology, project administration, supervision, writing – review and editing*: Omid Zehtabvar. *Formal analysis, resources, software, writing – original draft, writing – review and editing*: Seyyed Hossein Modarres Tonekabony. *Resources*: Iman Memarian.

## CONFLICT OF INTEREST STATEMENT

I and other writers promise that we have no interest in the results of this project, and we have avoided presenting any results in line with our interests.

## FUNDING INFORMATION

No financial aid was received for this study.

### PEER REVIEW

The peer review history for this article is available at https://publons.com/publon/10.1002/vms3.1213.

## ETHICS STATEMENT

The experiment was approved by the Animal Ethics Committee of the Faculty of Veterinary Medicine, university of Tehran, Iran.

## Data Availability

The data that support the findings of this study are available from the corresponding author upon reasonable request.
